# The Challenges of Putting Systems Thinking into Practice Comment on "What Can Policy-Makers Get Out of Systems Thinking? Policy Partners’ Experiences of a Systems-Focused Research Collaboration in Preventive Health"

**DOI:** 10.34172/ijhpm.2020.92

**Published:** 2020-06-11

**Authors:** John Boswell, Janis Baird, Ravita Taheem

**Affiliations:** ^1^Politics and International Relations, University of Southampton, Southampton, UK.; ^2^Human Development and Health, University of Southampton, Southampton, UK.

**Keywords:** Systems Thinking, Prevention, Public Health, Health Policy

## Abstract

In theory, ‘systems thinking’ offers a remarkably attractive solution to the persistent challenges of preventive public health. Haynes and colleagues’ recent analysis of the Prevention Centre in Australia offers reason for optimism that it might be possible to translate this promise into action on the ground. In this commentary, we critically assess the claims from this promising case study and their broader applicability to the cause of preventive health. We argue that, in many other contexts, persistent obstacles remain, such as a lack of buy-in from senior policy actors, and a lack of tangible or concrete action following through on an abstract commitment to systems thinking.

In theory, ‘systems thinking’ offers a remarkably attractive solution to the persistent challenges of preventive public health. In this view, interventions to improve public health should no longer be conceptualised, designed and implemented as isolated projects. Instead, systems thinking encourages public health academics and professionals to consider interrelationships with other projects, programme and policies across sectors.^1,2^ The goal is to practice joined-up interventions that better anticipate political and administrative obstacles and barriers to success, and leverage opportunities for reinforcing feedback from other activities. As something of a new orthodoxy in public health scholarship, studies mapping complex public health systems,^3^ and tracing positive and negative feedback loops in public health settings,^4^ are becoming ever more sophisticated.

In practice, however, the influence of ‘systems thinking’ has not yet been so profound for many public health policy-makers and professionals on the ground. Many remain confused by the implications, or sceptical about the utility in the messy ‘real world’ in which they are expected to apply these ideas. In this context, the study of Haynes et al is especially refreshing and informative.^5^ They offer one of the first detailed looks inside the world of systems thinking in public health practice. It is an exploration that offers some promising insights, but one that also appears to reinforce longstanding trade-offs and tensions in the pursuit of more systemic public health policy.

## Putting Systems Thinking Into Practice

Haynes et al richly detailed study of the Prevention Centre in Australia conforms to the highest standards of practice in qualitative research. It captures a rare, paradigmatic case of a significant institutional commitment to systems thinking in public health. Data collection is based on repeated interviews with 18 policy-makers working at different levels of seniority across a range of geographical areas. It is clear that the researchers built up a rapport of deep engagement and mutual trust, eliciting rich and vivid reflections on the topic at hand. Moreover, their commitment to multi-coder analysis ensures that the thematic insights are robust and rigorous.

Substantively, the study charts how a group of policy-makers and practitioners working at different levels from the Commonwealth government and across the States and Territories came together to co-produce a shared strategic vision for systemic public health. Their rich qualitative insights chart how, in spite of some reluctance or cynicism on the part of policy-makers, most seem to have developed a new or more rigorous appreciation for the value of systems thinking. They present the Prevention Centre as a model for policy learning – the sharing of experiential insights and accumulation of practical wisdom which enables them to understand the challenges they face in a new way. Haynes et al conclude of their participants that engagement with the Prevention Centre ‘shifted the way they think.’

Notwithstanding these positive developments, however, we think there is equally an opportunity to pause and reflect on the findings from this seeming ‘best case scenario’ for systems thinking in public health. We focus our critical discussion on two key elements. One zeroes in on the question of ‘whose thinking’ shifts. The other zeroes in on the gap between thought and action. The commentary is based both on a critical reading of the paper, and on our own experiences of systems thinking initiatives, especially in the United Kingdom, under far more challenging and resource-constrained institutional conditions than apparent in the Prevention Centre case. To be clear, we do not engage in this discussion to call into question the value of this paper – which, as we have said, offers a rare and immensely valuable insight into a systemic approach to public health in practice. Instead, we do so to join Haynes et al in further pushing along discussion about the challenges of translating new systems science into the real world of public health policy.

## But Who Is Really Shifting to Systems Thinking?

From the Prevention Centre case, we can see some promising signs for the broader goal of a more systemic view of public health. Interestingly, for example, Haynes et al show that the systemic view new to many in public health scholarship is not so new to policy actors on the ground. For many of them, it is intuitive; it accords with their own lived experience of pursuing projects and programmes in a complex administrative, political and social context. As such, there is clear appetite – evident especially among the rank-and-file public health policy-makers and practitioners in Haynes and colleagues’ account – for a more systemic approach to public health strategy. Yet there are two notable exceptions or omissions in the story, which evidence from elsewhere suggests could remain significant barriers to transformative change.

One is that senior officials interviewed in Haynes et al appear the most reticent to embrace systems thinking in a wholehearted way. Their reticence is consistent with scholarship on the challenges of leading a more systemic approach to public health focused on upstream prevention. Political and administrative leaders are faced with difficult trade-offs, immediate and pressing concerns and strictly routinised procedures of evaluation and accountability.^6,7^ In this context, it is hardly surprising that many find it challenging to take the ‘leap of faith’ to invest in a genuinely systemic approach in practice. This is important because the success of any co-production process may be determined by who engages (in spirit as well as in body). Engaging those at an operational level but without the power to change elements in the system may be a missed opportunity without authentic buy-in from high-level decision-makers with the power to enact change.

Two is that enthusiasm for systemic thinking in the Prevention Centre appears to remain limited to those already engaged in public health policy. Systems science emphasises the complex reciprocal relationships between different actors at different levels across cultural, economic and environmental processes and factors.^8,9^ In the case of the Prevention Centre, it is clear that the health policy-makers’ engaged were motivated by professional development, building capacity and the opportunity to engage with researchers. (Public health is a fairly academic discipline even for practitioners). However, collaboration between actors across sectors and covering broad areas of expertise is considered central to systems thinking.^10^ Also considering the implication of changes outside the intended jurisdiction of health policy is crucial to capture potential unintended consequences.^11,12^ As Haynes et al acknowledge, not having other sectors’ views to help understand the problem in environmental, economic, educational contexts seems a missed opportunity here.

## And What About the Gap Between Thought and Action?

The second key concern surrounds the practical impact of any ‘shift in how they think.’ Of course, the case of the Prevention Centre offers some promising signs. As evidence from elsewhere shows, describing the problem and developing a collective understanding of the issue is considered important in terms of challenging any assumptions made.^12,13^ Moreover, Haynes et al are quick to acknowledge and dwell on the frustrations of their interviewees about the lack of tangible progress. This finding reinforces the perennial conflict between policy and public health practitioners’ (elusive) search for “certainty” about the evidence surrounding any narrow intervention, and the commitment to a wide-angled, complex, adaptive approach that underpins an authentic systemic view.^14-16^ In this vein, Haynes et al report complaints about abstract ideas or intricate mapping exercises that only serve to complicate issues rather than provide obvious or clear solutions for policy-makers.

Nevertheless, we see a potentially bigger gap between thought and action than Haynes and colleagues’ overarching interpretation suggests – one which raises more fundamental challenges for implementing systems thinking on the ground. It is easy to adopt the ‘soft’ tools of systems thinking, and give the appearance of taking this view seriously.^17^ In the case of the Prevention Centre, we see a significant investment resulting in agreement to a set of meetings with diverse stakeholders, commitment to a shared approach to mapping and understanding the problem, the production of abstract strategy documents. Adopting any ‘hard’ tools, however, is rather more of a challenge for actors looking to grasp on to tangible policy instruments whose impact they can evaluate. One notable success on this front in the Prevention Centre case is the commitment to longer evaluation timescales, commensurate with a more complex and long-term view of cause and effect. But even in this very conducive political and administrative environment, it is much less clear that anyone involved – least of all the senior officials with overall accountability – has moved very far beyond the project orientation or emphasis on input-output measurement that typifies public health intervention. We can even detect some of this slippage in the data Haynes et al report where, for instance, a high-level policy-maker speaking warmly about the implications of systems thinking appears to be equating it to mathematical modelling. It begs the question of what sort of tangible impact we might expect out of other programmes and jurisdictions with much less of an investment in people, enthusiasm and resources than the Prevention Centre in Australia.

## Conclusion

Previous research has highlighted that a top down command and control approach is not effective in systems thinking. However, centralised government organisations have an important role in facilitating transdisciplinary work.^10^ The study by Haynes et al demonstrates the potential for policy-makers and researchers to apply systems approaches to public health problems. However, even under the optimal circumstance of the Prevention Centre, commitment to a systems approach did not always translate into effective practice. In the United Kingdom, local government public health teams have been tasked with leading the way on a whole systems approach to tackle obesity.^18^ This is against a backdrop of reduced funding, competing priorities and minimal training to implement the approach – circumstances that are far from optimal for effective multi-disciplinary working. Given health policy does not operate in a vacuum, consideration should be given to the conditions which can facilitate systems thinking in the context of government organisations. Research on the implementation of policy change in a complex healthcare system highlighted that different policy actors have different priorities and react based on their knowledge and interpretation of a given situation.^19^ This reiterates the value of collaboration across groups. Often getting buy-in from different departments, sectors, and political leaders in itself requires commitment and resource. Therefore issues such as relationships and power dynamics between groups in government settings and understanding how they change over time may be key in optimising these conditions.^20^ In addition training actors beyond public health policy-makers may be important to maximise the use of systems thinking to address complex public health challenges.

## Ethical issues

Not applicable.

## Competing interests

Authors declare that they have no competing interests.

## Authors’ contributions

All authors contributed equally to all parts of the manuscript.

## Authors’ affiliations

^1^Politics and International Relations, University of Southampton, Southampton, UK. ^2^Human Development and Health, University of Southampton, Southampton, UK.
